# A phantom study to evaluate three different registration platform of 3D/3D, 2D/3D, and 3D surface match with 6D alignment for precise image‐guided radiotherapy

**DOI:** 10.1002/acm2.13086

**Published:** 2020-11-12

**Authors:** Hsiang‐Chi Kuo, Michael M. Lovelock, Guang Li, Åse Ballangrud, Brian Wolthuis, Cesar Della Biancia, Margie A. Hunt, Sean L. Berry

**Affiliations:** ^1^ Medical Physics Department Memorial Sloan Kettering Cancer Center New York NY USA; ^2^ Radiation Oncology Department Norwalk Hospital Norwalk CT USA

**Keywords:** IGRT, image registration, target registration error

## Abstract

**Purpose:**

To evaluate two three‐dimensional (3D)/3D registration platforms, one two‐dimensional (2D)/3D registration method, and one 3D surface registration method (3DS). These three technologies are available to perform six‐dimensional (6D) registrations for image‐guided radiotherapy treatment.

**Methods:**

Fiducial markers were asymmetrically placed on the surfaces of an anthropomorphic head phantom (n = 13) and a body phantom (n = 8), respectively. The point match (PM) solution to the six‐dimensional (6D) transformation between the two image sets [planning computed tomography (CT) and cone beam CT (CBCT)] was determined through least‐square fitting of the fiducial positions using singular value decomposition (SVD). The transformation result from SVD was verified and was used as the gold standard to evaluate the 6D accuracy of 3D/3D registration in Varian’s platform (3D3DV), 3D/3D and 2D/3D registration in the BrainLab ExacTrac system (3D3DE and 2D3D), as well as 3DS in the AlignRT system. Image registration accuracy from each method was quantitatively evaluated by root mean square of target registration error (*rmsTRE*) on fiducial markers and by isocenter registration error (*IRE*). The Wilcoxon signed‐rank test was utilized to compare the difference of each registration method with PM. A *P* < 0.05 was considered significant.

**Results:**

*rmsTRE* was in the range of 0.4 mm/0.7 mm (cranial/body), 0.5 mm/1 mm, 1.0 mm/1.5 mm, and 1.0 mm/1.2 mm for PM, 3D3D, 2D3D, and 3DS, respectively. Comparing to PM, the mean errors of *IRE* were 0.3 mm/1 mm for 3D3D, 0.5 mm/1.4 mm for 2D3D, and 1.6 mm/1.35 mm for 3DS for the cranial and body phantoms respectively. Both of 3D3D and 2D3D methods differed significantly in the roll direction as compared to the PM method for the cranial phantom. The 3DS method was significantly different from the PM method in all three translation dimensions for both the cranial (*P* = 0.003–*P* = 0.03) and body (*P* < 0.001–*P* = 0.008) phantoms.

**Conclusion:**

3D3D using CBCT had the best image registration accuracy among all the tested methods. 2D3D method was slightly inferior to the 3D3D method but was still acceptable as a treatment position verification device. 3DS is comparable to 2D3D technique and could be a substitute for X‐ray or CBCT for pretreatment verification for treatment of anatomical sites that are rigid.

## 1 INTRODUCTION

Modern radiotherapy (RT) demands precise and accurate treatment delivery. The mechanical accuracy of linear accelerators (LINACs) in conjunction with advanced technologies such as high‐quality imaging systems and six degree of freedom (6DoF) robotic couches have produced RT systems capable of delivering dose with submillimeter accuracy. Among all these technologies, image registration plays an important role in ensuring that the spatial accuracy of dose delivery falls within clinical tolerances. It is essential to understand the uncertainty associated with the registration produced by a given imaging device. AAPM Task Group (TG) report 132^1^ has reviewed current approaches and solutions for image registration in RT and has made recommendations for quality assurance and quality control of the clinical processes using these imaging devices. In an image registration used for image‐guided radiotherapy (IGRT), the reference image is usually the planning computed tomography (CT) simulation scan. This is registered with images acquired immediately prior, and during, dose delivery. Corrections to the position of the target and critical structures derive from the registration of these images with the reference image. There are different imaging devices available in the treatment room to verify the treatment position in three dimensions (3D), for example, paired planar kV X‐rays, cone beam computed tomography (CBCT), and optical surface imaging (OSI). All have unique design characteristics which influence the performance of the resulting image registrations.

All registration methods discussed here result in rigid body transformations. There are different approach of 2D3D registration.^2^ The most popular one is the intensity‐based method which is the one compared in this study. The (2D3D)^2^, ^3^ registration process compares the intensity of the paired planar kV X‐ray images with the intensity of digitally reconstructed radiographs (DRRs) created from the planning CT. A similarity metric is minimized iteratively to optimize the translations and rotations used to generate the DRRs by minimizing the geometrical difference between the paired DRRs and the paired X‐ray image. 3D3D method directly compare two volumetric data set (KV‐CBCT and planning CT in this study) and compute the geometric transformation of the two volumetric images by minimizing the similarity metric (intensity difference, mutual information, etc.) in three dimensions. Optical surface imaging renders the object surface in image space. The geometric transformation is determined by minimizing the distance of the correspondence (most popularly using Integrative Closet Point method) in the two three‐dimensional point set sampled from in‐room active illuminated projector/receiver (typically, structured‐light/camera) and the planning CT surface (3DS). The principle of structured‐light 3D surface imaging techniques is to extract the 3D surface shape based on the information from the distortion of the projected structured‐light pattern.^4^ To keep a reasonable frame rate for real‐time monitoring, the point cloud is restricted to a limited and predefined region of interest (ROI).

All these modalities have large numbers of points, pixels, or voxels to be matched and rely on an iterative optimization procedure to search for the geometrical transformation. In addition to the uncertainties due to the spatial resolution of both image sets, image artifacts, noise, and possible effects due to preprocessing alterations to correct for the nonuniform intensity distribution between two images, iterative methods normally cannot guarantee that a solution converges to a global minimum and are not as robust as analytical methods.

Image registration based on extrinsic markers is straightforward and does not require complex optimization algorithms. For rigid bodies, the point match (PM) problem is typically defined to be the problem of finding the translation vector and the rotation matrix that produces the least‐square fit of the corresponding fiducial points. The problem of determining the rotation matrix can be reduced to the “Orthogonal Procrustes Problem,”^5^ which has many closed form solutions.^6^

Assuming the points are not colinear, the limitation of PM is the localization error of the individual markers.^7^ Increasing the number of fiducial points reduces the localization uncertainty. One‐millimeter target registration error is theoretically possible when using four extrinsic fiducials with 2‐mm CT scan slice thickness.^8^ PM‐based registration using extrinsic markers is accurate and is therefore often used as a ground truth for validation of other registration methods under rigid conditions. It is the aim of this study to compare the accuracy of several image guidance systems used in RT using PM as a baseline.

## 2 MATERIALS AND METHODS

### 2.A Data acquired

Registration accuracy was studied in two clinical cases: the cranium and the torso. Thirteen external markers were asymmetrically placed around the surface of a cranial phantom (STEEV) (CIRS Inc., Norfolk, VA). Eight of the markers (the “C” group) were used to compute the PM transformation (using the singular value decomposition — SVD‐based root mean square scheme in the following section) between two set of images. The remaining five markers (the “V” group) were used to validate and compare the registration accuracy of all from different registration methods. Similarly, eight markers were asymmetrically placed around the surface of a body phantom (ET verification phantom, BrainLab, Munich, Germany). Five of the markers (“C” group) were used to compute the transformation for PM method. The remaining three markers (“V” group) were used to validate and compare all the registration methods in this study.

Planning isocenters were placed, near the splenium of the corpus callosum and in the right posterior cerebellum of the cranial phantom, and at the mid‐body above the L5 lumbar vertebral body of the body phantom. Setup verifications were performed on (a) CBCT acquired from the on‐board imager (OBI) (Truebeam and 21EX, Varian Medical Systems, Palo Alto, CA), (b) paired planar X‐rays from the ExacTrac system (BrainLab, Munich, Germany), and (c) OSI acquired from the AlignRT system (VisionRT, London, UK). The phantoms were setup on a baseplate with random rotations in three axes (Yaw, Roll, and Pitch). All rotations (different combination of Pitch, Yaw, and Roll) were restricted to be within ±3 degrees. A total of 36 different setups, 20 in the cranial phantom and 16 in body phantom, were studied and performed both at TrueBeam (“TB” group) and at 21EX (“EX” group). Number of registration and the resolution applied in each technique were summarized in Table 1.

**Table 1:** Number of registration and resolution of the images taken for the technique applied in this study.

Technique		PM	3D3D	2D3D	3DS
Number	cranial/body	(20/16) × 2^a^	(20/16) × 2^b^	20/16	20/16
Resolution cranial/body (mm)	Planning CT	0.6 × 0.6 × 1.25^c^/0.8 × 0.8 × 2	0.6 × 0.6 × 1.25/0.8 × 0.8 × 2	0.6 × 0.6 × 1.25/0.8 × 0.8 × 2	0.6 × 0.6 × 2/0.8 × 0.8 × 2
	CBCT	0.7 × 0.7 × 1/0.9 × 0.9 × 1	0.7 × 0.7 × 1/0.9 × 0.9 × 1	0.7 × 0.7 × 1/0.9 × 0.9 × 1	
	X‐ray image			0.6	

^a^ 20/16 performed at TB, PM_TB_; 20/16 performed at EX, PM_EX_.

^b^ 20/16 performed at Varian platform‐3D3DV; 20/16 performed at ExacTrac platform‐3D3DE.

^c^ cranial planning CT for TB used 1.2‐mm slice thickness, cranial planning CT for EX used 2‐mm slice thickness.

### 2.B Rigid body transformation: Least‐square solution using SVD

Assume there exist two corresponding point sets represented by Matrices A and B. The question is to determine a rotation matrix R and a translation vector T using a least‐squares scheme:

(1) $$\min_{R\in \Omega }{∥RA^{t}+T‐B^{t}∥}^{2}$$

where *A^t^* and *B^t^* denote the transpose of the matrix *A* and *B*, respectively, and Ω is a set of 3 × 3 orthogonal rotation matrices such that

(2) $$\Omega =\left\{R|R^{t}R={\text{RR}}^{t}+I_{3};\;\det\left(R\right)=1\right\},$$

where R^t^ denotes the transpose of the matrix R.

Using the SVD solution developed by Arun et al. in 1987,^8^ Soderkvist^6^ outlined the algorithm in the following steps:

1. Compute the centroids of the point sets A and B, $A_{c}$=$\frac{1}{n}\sum_{1}^{n}A_{i}$ $,B_{c}$=$\frac{1}{n}\sum_{I}^{n}B_{i}$,
2. Re‐center A points and B points to the centroids, $A^{\prime }=A‐A_{c}$, $B^{\prime }=B‐B_{c}$,
3. B' = RA', the original problem becomes $\min_{R\in \Omega }{∥R{\left(A^{\prime }\right)}^{t}‐{\left(B^{\prime }\right)}^{t}∥}^{2}$,
4. Compute SVD of B’, B’=UΛV^t^, the optimal rotation matrix, R, that maximizes the desired trace is R = VU^t^, where VV^t^ = UU^t^ = I_3_,
5. T = B^t^ − RA^t^.

The following image registration techniques were investigated and validated using the extrinsic fiducial makers: (a) an in‐house PM image registration program using the above SVD scheme applied to the fiducial markers localized from planning CT and CBCT images, (b) 3D3D image registration methods performed on Varian’s platform (3D3DV) and ExacTrac platform (3D3DE) to compare CBCT to planning CT, (c) a 2D3D image registration method performed on the ExacTrac platform to compare paired X‐ray images back to the planning CT, and (d) a 3DS image registration method performed on an AlignRT platform to compare alignment of the surfaces rendered from the planning CT and OSI. Using the TG‐132 report’s classification, the features of each image registration technique are summarized in Table 2.

**Table 2:** The important features of images registration methods applied in this study.

	PM	3D3D	2D3D	3DS
Dimensionality	3D	3D	3D; 2D	3D
Nature of registration	Extrinsic; Feature based	Intrinsic; Intensity based	Intrinsic; Intensity based	Intrinsic; Feature based
Nature of transformation	Rigid Transformations in 6D	Rigid Transformations in 6D	Rigid Transformations in 6D	Rigid Transformations in 6D
Interaction	Interactive	Automatic	Automatic	Automatic
Optimization procedure	Analytic Solution	Iterative	Iterative	Iterative
Modalities involved	CT; CBCT	CT; CBCT	CT; Planar X‐ray	CT; OSI
Subject	Same	Same	Same	Same
Limitation	Localization; Number of markers	Resolution; Image quality; Robustness of optimization	Resolution; Image quality; Robustness of optimization	Resolution; ROI; Robustness of optimization

### 2.C Validation and evaluation

Image guidance system based on fiducial registration usually display the measure of registration accuracy based on the goodness of fit of the fiducials. Using fiducial markers with rigid geometry, the true transformation from one CT image set to another CT image set should register the two image sets with a residual error of zero. Fiducial registration error (FRE), which is a common measure of goodness of fit, is the distance between corresponding fiducial points after registration. The problem of registration reduces to finding a rotation and translation that minimizes FRE. For C group with n fiducial markers,

(3) $$\text{FRE}=∥Ra_{i}+T‐b_{i}∥,$$

where *a_i_* ∈ A and *b_i_* ∈ B.

The accuracy of marker registration is limited by the fiducial localization error (FLE) and the number of the noncollinear fiducial maker (n) used to compute the transformation. FLE can be calculated by FRE using the approximation derived by Sibon,^9^

(4) $$〈{\text{FLE}}^{2}〉=\sqrt{\frac{n}{n‐2}}*〈FRE^{2}〉,$$

where < .> means “expected value of.”

To assess the accuracy of all registration techniques, an independent group (V) of fiducial markers was used to compute the target registration error (TRE), which is the distance between corresponding markers not used in generating the registrations. For the V group with m fiducial markers,

(5) $$\text{TRE}=∥Ra_{j}+T‐b_{j}∥,$$

where *a_j_* ∉ A and *b_j_* ∉ B.

A root mean square target registration error (*rmsTRE*) was calculated for each setup observation to compare the accuracy of the registration techniques applied in this study.

(6) $$rmsTRE=\sqrt{\frac{1}{m}\sum_{j=1}^{m}{\left(Ra_{j}+T‐b_{j}\right)}^{2}}$$

where *R* and *T* are the values calculated by PM, 3D3DV, 3D3DE, 2D3D, and 3DS methods, respectively.

**Fig. 1:**
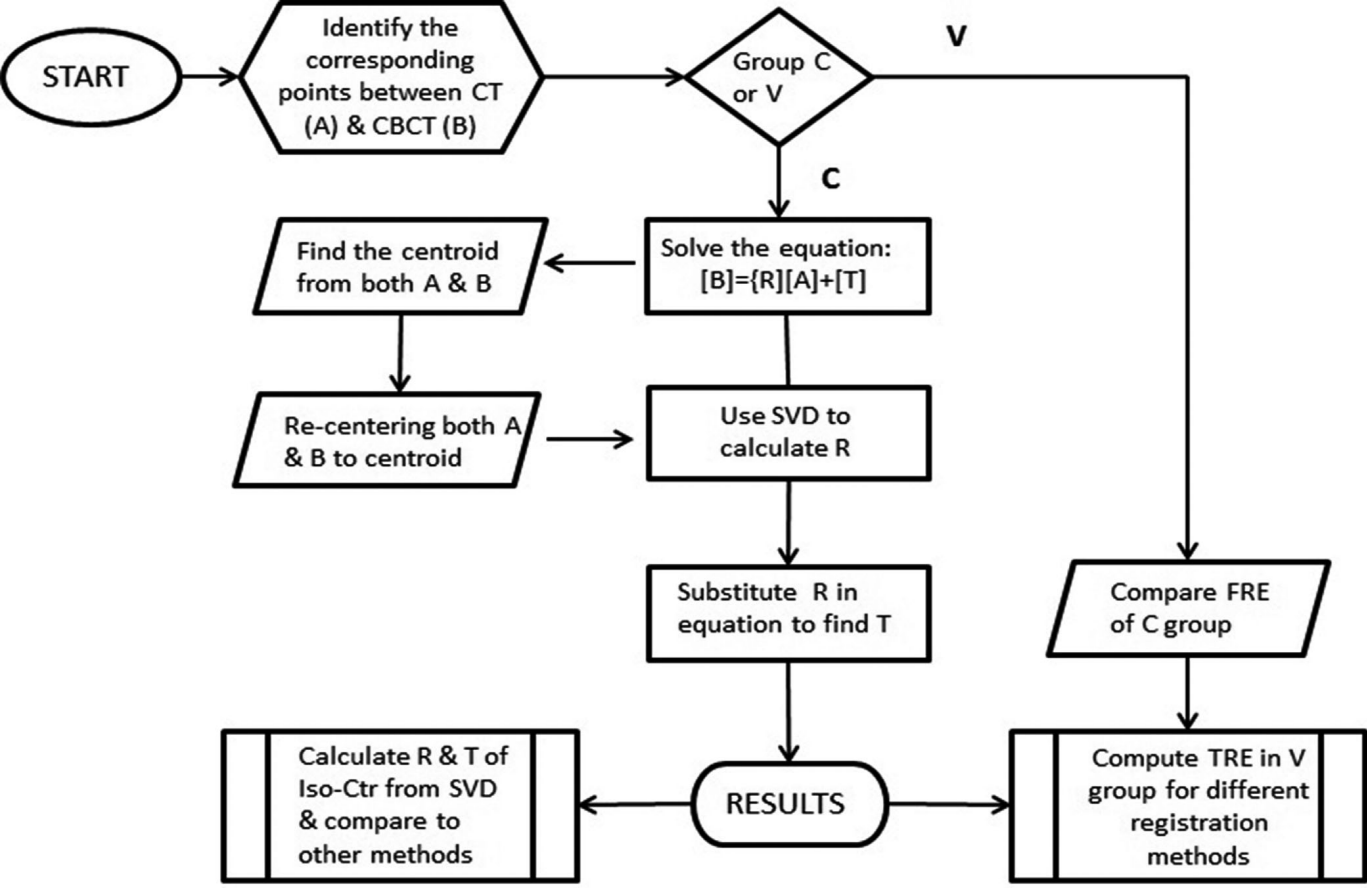
The flowchart of the algorithm of the least‐square solution using SVD and the verification process to compare different image registration methods.

The extrinsic markers were easy to place and to study the known transformation from one image to another image. However, they were placed outside of the phantom and were distant from the treatment target. The centroid of the extrinsic makers is typically close to the treatment target (as in this study) such that the measured error of the marker after registration has larger impact from the rotation error than the treatment target. Internal treatment target is clinically close to the Iso‐Center (ISO). Modern IGRT technique combined with 6DoF couch has been able to shift the residual error between the positions in the verification image to the position in the planning image with transformation referenced to ISO. Using the results of the PM method as ground truth, the residual error of ISO setup correction (*IRE*) was calculated by comparing the ISO transformation in 6D (3D translation and 3D rotation) returned from the commercially available image registration in each setup to the ISO transformation calculated from the PM method. Figure 1 diagrams the method applied in this study.

### 2.D Statistics

Wilcoxon signed‐rank test was utilized to compare the difference of each registration method with the PM method. A *P* < 0.05 was considered significant.

## 3 RESULTS

**Fig. 2:**
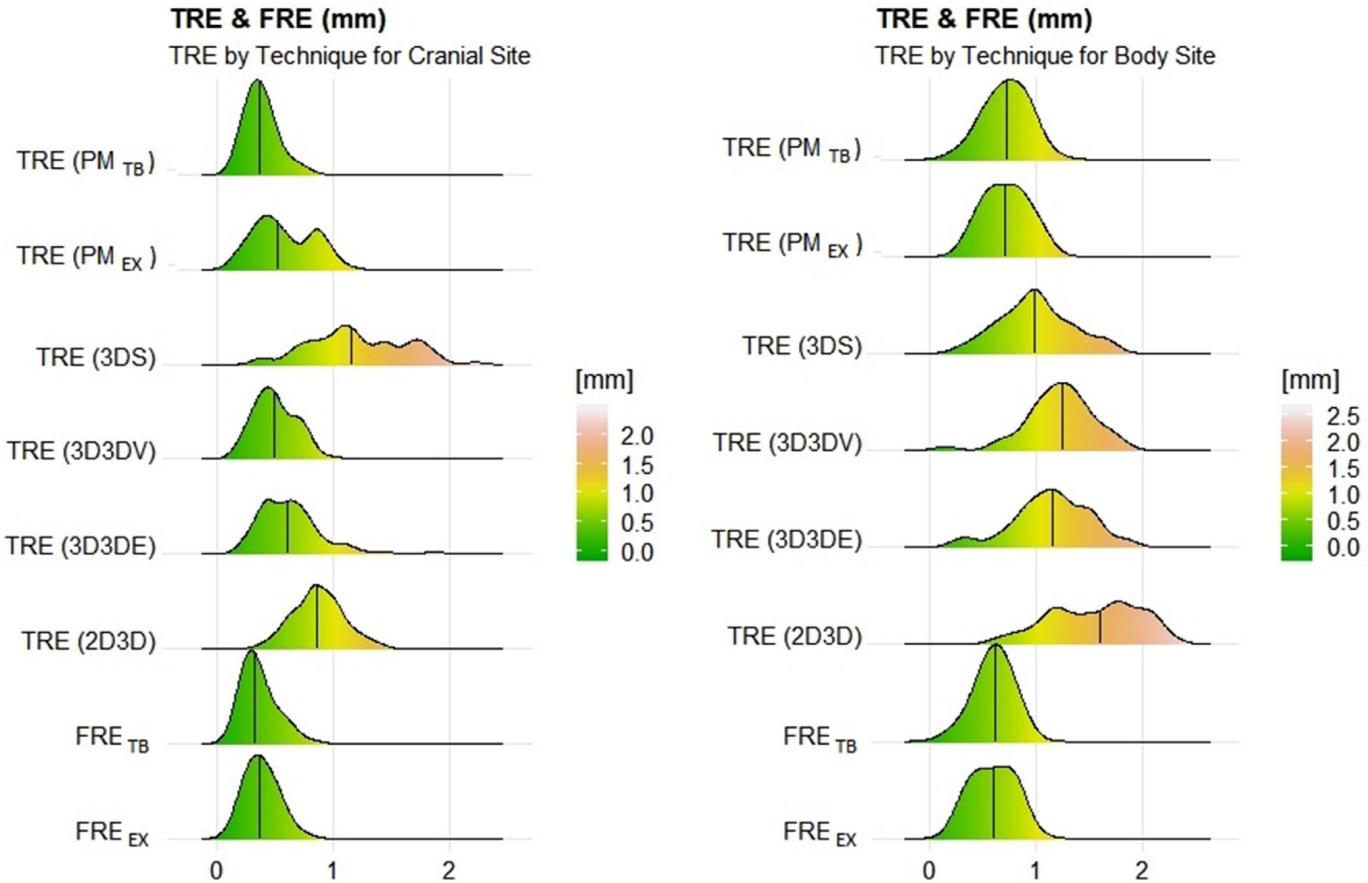
Probability distribution of TRE, FRE, and all other image registration methods by site. Plots were scaled such that the area under each plot is 1, such that the total number of counts is equivalent to the total number of observations.

Figure 2 displays the probability distribution of the registration error for each individual marker point. For the FRE analysis, there were 132 match points in the cranial case and 80 match points in the body case; for the TRE analysis, there were 80 match points in the cranial case and 48 match points in the body case. The FRE plots are approximately Gaussian distributed and had mean values of 0.4 and 0.6 mm for cranial and body, respectively. The peak of the FRE plot was relative flat in 21EX, which could be due to the image quality and isocenter accuracy at TrueBeam are better than those at 21EX.

Using *rmsTRE* as evaluation metric, the accuracy of each image registration method in this study was summarized in Table 3. Overall, the PM method applied in this study had the smallest mean and standard deviation (STD) and was considered the most accurate. The mean value of *rmsTRE* using 3D3D was approximately 0.1–0.5 mm higher than the PM method and was close to half of the maximum pixel dimension. The mean value of *rmsTRE* using 2D3D and 3DS was found to be 0.3–0.8 mm higher than the PM method.

**Table 3:** Summary of the mean ± SD *rmsTRE* of the image registration methods applied at cranial and body sites.

	*rmsTRE*	
	Cranial (N = 20)	Body (N = 16)
PM_TB_	0.4 ± 0.1 mm	0.7 ± 0.1 mm
PM_EX_	0.6 ± 0.1 mm	0.7 ± 0.1 mm
3D3DV	0.5 ± 0.1 mm	1.2 ± 0.2 mm
3D3DE	0.6 ± 0.2 mm	1.1 ± 0.3 mm
2D3D	0.9 ± 0.2 mm	1.5 ± 0.1 mm
3DS	1.2 ± 0.2 mm	1.0 ± 0.1 mm

**Fig. 3:**
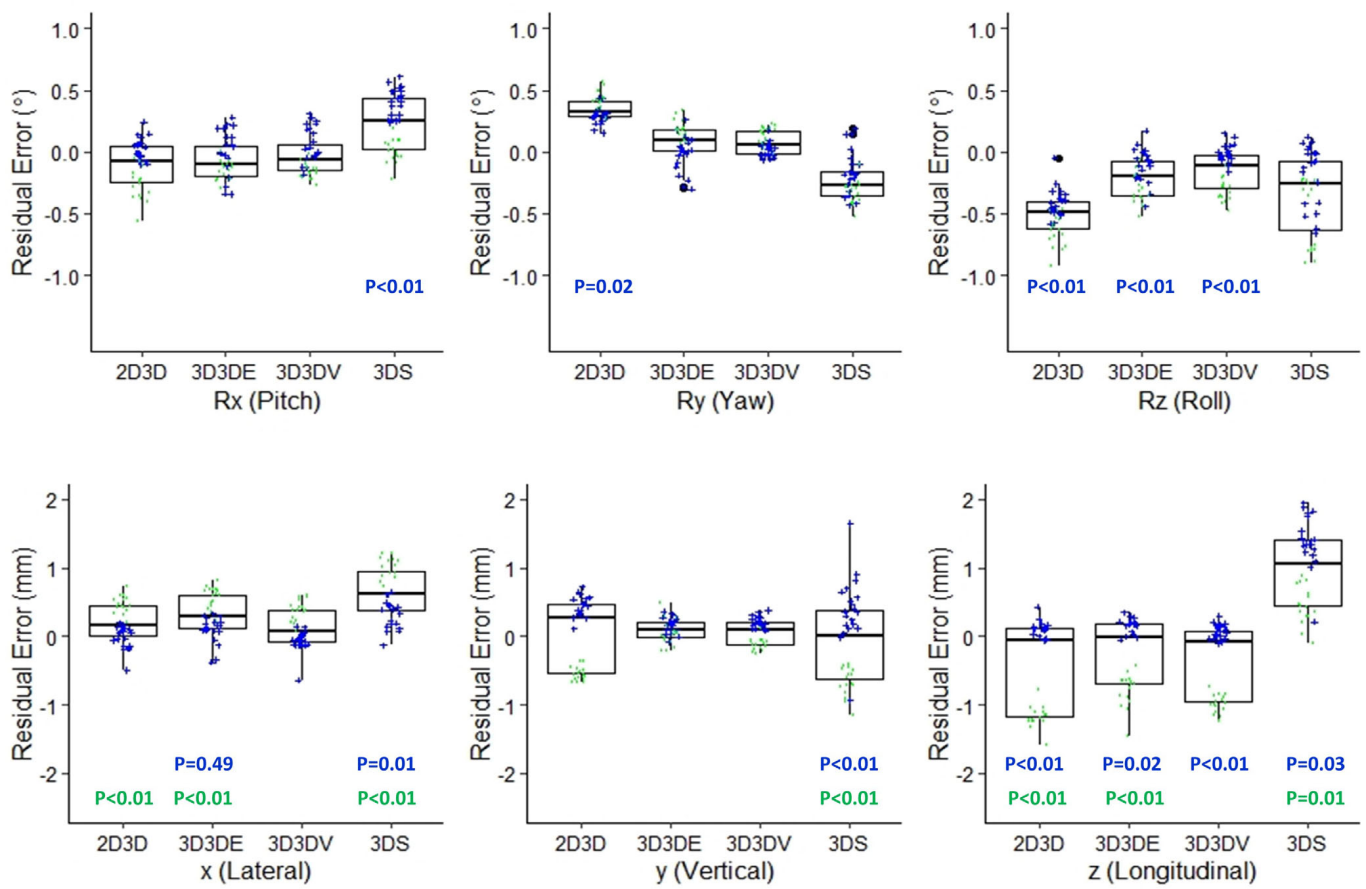
Box plot comparison of *IRE*in 6D. Rx, Ry, and Rz are rotation along couch lateral, vertical, and longitudinal, respectively. The error distribution of both cranial (+, blue color) and body (·, green color) are shown on the same plot.

Figure 3 is a box plot with jittered points populated to show the distribution of the *IRE* by technique and site, in 6D. The residual error was clustered at different locations for the cranial and body phantoms. The clustering feature represents the precision of the multiple experiments in this study from the individual subject (site or phantom). If more sites or more subjects were included in this study, the mean error for the population should be close to zero as long as there is no significant systematic error (e.g., image calibration error). Including both sites under the same box plot, the 3D3D method was the best among the three methods in terms of the mean and the range of the residual error. The range of the error increased more for the 3DS method when evaluating both sites together, especially the y (depth) and Rz (Roll) dimensions. Wilcoxon rank test with *P* < 0.05 were shown in the plots.

**Fig. 4:**
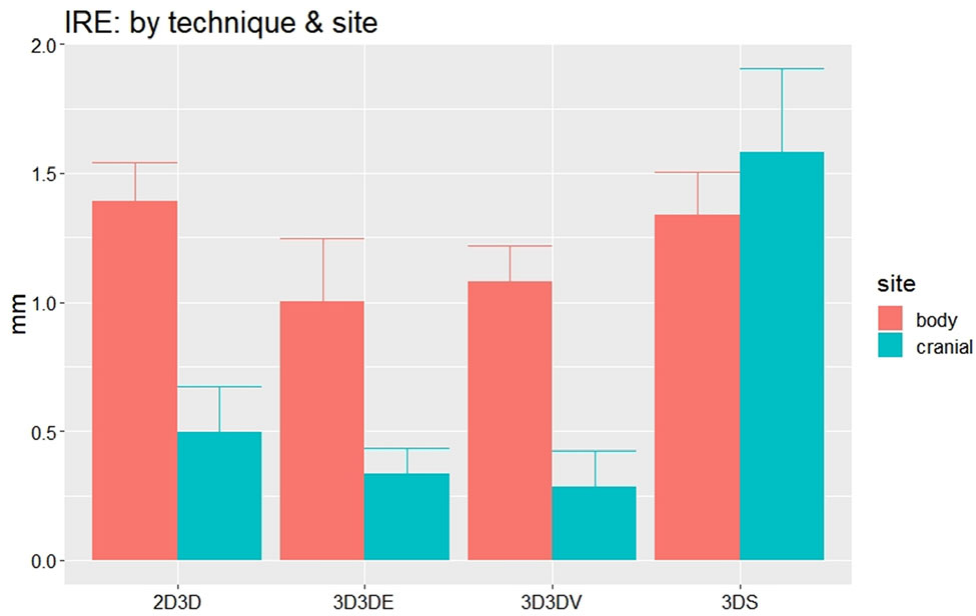
Mean and one SD range of *IRE*by technique and by site.

Figure 4 displays the magnitude of the *IRE* for each technique compared to the PM method. They were 0.3 mm/1 mm for 3D3D, 0.5 mm/1.4 mm for 2D3D, and 1.6 mm/1.35 mm for 3DS for the cranial and body phantoms respectfully.

## 4 DISCUSSION

To compare the accuracy of different IGRT techniques used for rigid body transformations, access to the geometric transformation is required (TG‐132).^1^ The point‐based technique using implanted makers quantitatively evaluates registration accuracy using TRE which can be used to compare the accuracy of multiple registration methods. This study is the first one using extrinsic makers as a reference to evaluate widely available image registration methods used for modern IGRT. The PM method using SVD is one of the closed form solutions of the orthogonal procrustes problem and has been shown to have marginally better accuracy and stability than other closed form solutions.^6^ By placing the extrinsic markers nonuniformly on the phantom, the accuracy of the PM method should be largely dependent on the fiducial marker localization error (FLE). Using Eq. (4) derived by Fitzpatrick,^10^ FRE values were found to be 0.36–0.59 mm, depending on the pixel size. The corresponding FLE was between [0.4,0.75] mm. They were around one third the size of the largest pixel dimension. This FLE range is comparable with the values 0.66 ± 0.33 mm (CT resolution in the study, 0.41 × 0.41 × 2 mm) reported by Zhang et al.^11^

Unlike FRE, which does not depend on the configuration of the fiducial markers, the accuracy of PM method evaluated independently by *rmsTRE* is optimal when N (number of markers used to compute transformation) increases and the distance between makers is large.^10^, ^12^ The *rmsTRE* values of the PM method were slightly (0.1 mm) larger than the mean FRE, which was compatible with the results from literature.^13^ In addition to fairly small values of FLE, FRE, and *rmsTRE* of PM method, using the same V group markers to compare the image registration methods in this study, PM method had the smallest *rmsTRE*. These results validated that PM method could be used as a reference to evaluate the accuracy of the image registration methods of modern IGRT technique.

The two intensity‐based 3D3D methods evaluated in this study both showed similar mean and variation of *rmsTRE*. Comparing the ISO movement of the image from planning CT to CBCT, the *IRE* had a mean magnitude of 0.3 mm at cranial sites and 1.1 mm at body sites (Fig. 4). They were slightly better than the *rmsTRE* of the extrinsic fiducial markers which were placed on phantom surface and could have large impact from rotation error than the (ISO) point close to the rotation center.

Many studies have compared 2D3D‐paired X‐ray images to 3D3D CBCT in phantom or in patients.^14^, ^15^, ^16^, ^17^, ^18^ The typical improvements in 3D3D to 2D3D were at the order of millimeter. Our study showed that the intensity‐based 2D3D registrations had *rmsTRE* of 0.9 and 1.5 mm at cranial and body site, respectively. They were 100% larger than the PM method and were about 50% larger than the 3D3D method. The *IREs* were reduced to 0.5 and 0.9 mm for site cranial and body site, respectively, if comparisons were made at the ISO. In analyzing 30 setup verification on SRS cranial patient in our institute (2018 ~ 2020) comparing the results of 2D3D‐paired images to the results of 3D3D CBCT, the mean ± SD differences were 0.0 ± 0.3 mm, 0.1 ± 0.3 mm, 0.0 ± 0.3 mm at vertical, longitudinal, lateral direction, respectively; and were −0.4 ± 0.3°, 0.0 ± 0.3°, 0.4 ± 0.3° at Yaw, Pitch, roll direction, respectively.

3DS uses the same feature‐based registration as the PM method. The corresponding points are known and are limited for the PM method, but for the surface registration method, the point cloud needs an optimization process to find the correspondence and to perform the cloud alignment. Interactive closest point (ICP) is the most popular optimization technique^19^, ^20^, ^21^ for the point cloud registration; however, it is less robust compared to the PM method. The 3DS technique uses structured light^22^ which is a pseudorandom speckle pattern projected on an object surface and the distorted speckle pattern sensed by the camera is used to calculate the speckle point’s spatial position.^4^ It may be that both *rmsTRE* and *IRE* in this study were larger in the cranial case than the body case due to this speckle pattern’s discrete random point nature and when the sensing ROI is limited to the sharply varying slopes in the nasal area. Another result from this study showed that the *rmsTRE* was less than the *IRE* in both cranial and body sites. These results were different from the intensity‐based registration which performed evaluation based on the entire image. 3DS uses ROI to optimize 3D alignment, ROI normally is partially limited to the anterior surface and has its centroid distance away from ISO. An optimal match at the surface may not necessarily be an optimal match at deeper locations due to the limitations of iterative optimization. A minimal rotational mismatch will introduce translation error of point distance away from the rotation center.^6^ This may explain why a larger registration error (especially the translation) was observed at the ISO than at the maker points on surface.

Camera using in OSI system is sensitive to temperature. A clinical use of AlignRT should be operated under well‐controlled room temperature. It is a nonradiation method which acquires image under preset frame rate and can monitor treatment under real time. Comparing to the studies in the literature, the same surface imaging system has been shown to be accurate and precise to be within 0.5 mm/0.1° (translation/rotation) in the dynamic mode or tracking mode;^4^, ^23^, ^24^, ^25^ however, the absolute positional accuracies to the planning position compared to bony anatomy from CT or x‐ray were close to 1 mm/1° at head and neck^25^, ^26^ and body^4^ sites. Li’s study^27^ compared a group of cranial SRS patients receiving setup verification using AlignRT against CBCT setup. The patient‐specific residual shifts were between 1 and 2 mm. Using extrinsic fiducial markers as the gold standard and reporting using *rmsTRE*, this study found values of 1.2 mm/1 mm for cranial and body sites. The mean *IRE* using AlignRT was within 0.5° in all three‐rotation axis both cranial and body and was within 1 mm translationally except the longitudinal direction at the cranial site. Using a smaller CT slice thickness and a different ROI may improve the *IRE*. This warrants further study.

This study implemented a robust PM registration using extrinsic fiducial markers as a gold standard to assess the accuracy of image registrations applied in modern IGRT techniques. Studies were done under rigid phantom with the clinical application limited to treatment site which is rigid, for example, cranial, paraspinal, and pelvis bone. All the three IGRT platforms discussed in this study using auto‐registration process is optimal under rigid condition. Treatment sites with shape or position (relative to nearby bone) varies from day to day, their treatment accuracies can be enhanced by using different way, for example, using fiducial makers (prostate in 2D3D match),^28^ using a soft tissue mode by limiting a smaller ROI to the target (lung lesion in 3D3D match),^29^ using OSI to align setup (arm, breast, H&N) followed with X‐ray or CBCT, or using nonrigid registration followed with adapted plan (Adaptive Radiation Therapy).^30^ The accuracy of the nonrigid condition using the methods listed above is beyond the scope of this study. Although the PM results in this study were limited by the pixel size and the number of markers, the achieved TREs were small and the evaluation of the commercial image registration methods can be used as a guideline in clinical IGRT implementation.

## 5 CONCLUSIONS

The accuracy of the image registrations utilized in modern IGRT techniques has been evaluated using a PM method. 3D3D using CBCT had the best accuracy among all tested methods. It should be chosen as the gold standard in clinical practice for all treatment techniques. 2D3D method was slightly inferior to the 3D3D method and would be acceptable as a treatment position verification in cranial SRS case. 3DS is comparable to 2D3D technique and could be a substitute for X‐ray or CBCT for pretreatment verification for the treatment of anatomical sites that are rigid and are non‐SRS/SBRT treatments. The limitation of the study is that it was done under rigid condition. Clinical application under nonrigid condition should be applied carefully.

## CONFLICT OF INTEREST

The authors have no conflict of interest to disclose.
